# Emerging Perspectives of Bone Metastasis in Hepatocellular Carcinoma

**DOI:** 10.3389/fonc.2022.943866

**Published:** 2022-06-29

**Authors:** Xiaofeng Yuan, Ming Zhuang, Xi Zhu, Dong Cheng, Jie Liu, Donglin Sun, Xubin Qiu, Yunjie Lu, Kurt Sartorius

**Affiliations:** ^1^ The Third Affiliated Hospital of Soochow University, Changzhou, China; ^2^ Department of Infectious Diseases, The First Peoples’ Hospital of Kunshan, Kunshan, China; ^3^ Hepatitis Diversity Research Unit, School of Internal Medicine, University of the Witwatersrand, Johannesburg, South Africa; ^4^ Africa Hepatopancreatobiliary Cancer Consortium (AHPBCC), Mayo Clinic, Jacksonville, FL, United States; ^5^ School of Laboratory Medicine and Molecular Sciences, College of Health Science, University of KwaZulu-Natal, Durban, South Africa

**Keywords:** hepatocellular carcinoma (HCC), bone metastasis, biomarkers, osteolysis, bone remodeling

## Abstract

Recent evidence suggests the global incidence and mortality of hepatocellular carcinoma (HCC) are increasing. Although the highest incidence of HCC remains entrenched in WHO regions with high levels of HBV-HCV infection, the etiology of this disease is rapidly changing to include other lifestyle risk factors. Extrahepatic metastasis is a frequent feature of advanced HCC and most commonly locates in the lungs and bone. Bone metastasis in HCC (HCC-BM) signals a more aggressive stage of disease and a poorer prognosis, simultaneously HCC-BM compromises the function and integrity of bone tissue. HCC induced osteolysis is a prominent feature of metastasis that complicates treatment needed for pathologic fractures, bone pain and other skeletal events like hypercalcemia and nerve compression. Early detection of bone metastases facilitates the treatment strategy for avoiding and relieving complications. Although recent therapeutic advances in HCC like targeting agents and immunotherapy have improved survival, the prognosis for patients with HCC-BM remains problematic. The identification of critical HCC-BM pathways in the bone microenvironment could provide important insights to guide future detection and therapy. This review presents an overview of the clinical development of bone metastases in HCC, identifying key clinical features and identifying potential molecular targets that can be deployed as diagnostic tools or therapeutic agents.

## Background of Bone Metastasis in HCC

A recent analysis of the Globocan 2020 results indicates that the highest incidence of liver cancer persists in East Asia (17.8), North Africa (15.2), Micronesia (14.6), South East Asia (13.6) and Micronesia (11.4) against a global ASR of 9.5 (1). Hepatocellular carcinoma makes up > 75% of all primary liver cancer incidence (2, 3) and accounts for the fourth highest number of cancer-related death in the entire world (4). The incidence and etiology of HCC differ markedly across the WHO regions. In regions like Eastern Asia and Sub-Saharan Africa, for example, where HBV infection is endemic, the etiology of disease is significantly different from risk factors like NASH and NAFLD that predominate in Western countries. Early detection of HCC is also more likely in developed countries and patients in developing countries often only present at advanced stage disease (5, 6). Early diagnosis or screening for HCC is crucial and eliminating risk factors like viral infection (HBV/HCV), alcohol, dietary toxins, tobacco, aflatoxins and aristolochic acid is likely to reduce incidence more than current clinical treatment options. Due to progress in diagnostics of HCC such as CT/MRI, PET-CT and bone scintigraphy (BS), during the past 20 years, overall survival rates for HCC patients have improved (7–9). Nevertheless, extrahepatic metastases have become more common in recent years and most HCC patients are only diagnosed at an advanced stage. About 16.1% to 38.5% of HCC patients exhibit bone metastasis (BM) at first diagnosis, and 11.7% develop BM following curative resections (10). Patients with HCC who have developed bone metastasis (HCC-BM) have a poor prognosis and a median survival time of only 4.6 months. Risk factors contributing to poor survival among HCC-BM patients include Child-Pugh class A group, alpha-fetoprotein (AFP) levels (> 30 ng/mL), and tumor size (> 5 cm) (11). Moreover, the majority of HCC-BM patients suffer from skeletal-related events (SREs), including fractures and spinal cord compression caused by pathological conditions. Symptoms of these SREs include severe pain and neurological deficits that drastically deteriorate the patients’ quality of life. Sadly, there is no definitive therapeutic strategy for the management of HCC-BM. There are contrasting findings about the extent of HCC-BM and some studies indicate it is an infrequent form of presentation (10, 12) whereas others suggest it is relatively common and occurs in ~33.3% of patients with HCC (13). HCC cases reported with bone metastasis tend to have multiple metastatic spreads elsewhere as well (14).

In most cases, bone metastases from HCC are irreversible and incurable, as well as associated with pathological fractures, severe pain, decrease quality of life and poor prognosis (15). Bone metastases increase the burden on healthcare providers and individuals due to switching the medical paradigm from cure to palliation. As a result of advancements in the treatment of HCC-BM patients, it has become possible to identify which patients benefit the most from the treatments available (16). On a more encouraging note, the combined strategies of locoregional therapies and/or systemic therapy are constantly being evaluated. This review paper specially describes HCC-BM pathogenesis and identifies key biomarkers and potential therapeutic agents that could potentially be employed for the early diagnosis and/or treatment.

## Metastatic Dissemination in HCC

HCC pathogenesis occurs in a setting of chronic inflammation, fibrogenesis and liver damage against a background of frequent immune reactions, uncontrolled hepatocyte proliferation and the frequent mutations of proto-oncogenes and tumor suppressor genes (2). Typically, a key step in HCC metastasis is the loss of regulatory controls in cellular replication and metastasis occurs in conditions of chromosomal instability that can cause a leakage of tumor cell DNA that spread to distant organs (17). When primary HCC proliferates locally, epithelial-mesenchymal transformation (EMT) is activated by widespread epigenetic reprogramming of gene expression, and epithelial cells lose their cell polarity and adhesion properties, becoming more migratory and invasive (18). Once cell polarity transition occurs, polarized epithelial cells lose intercellular crosslinking and acquire a spindle-like morphology thus facilitating migration to distant organs (19). Simultaneously neo-angiogenesis is stimulated to deliver oxygen, nutrients, and growth factors to facilitate tumor growth and enable invasion (20). Vascular endothelial growth factor (VEGF), known as a crucial factor driving angiogenesis in the primary HCC lesion and its metastasis to bone, plays a critical role in stimulating bone resorption and facilitating tumor growth in bone (21).

### Macro-and Microinvasion in HCC-BM

**Figure 1:**
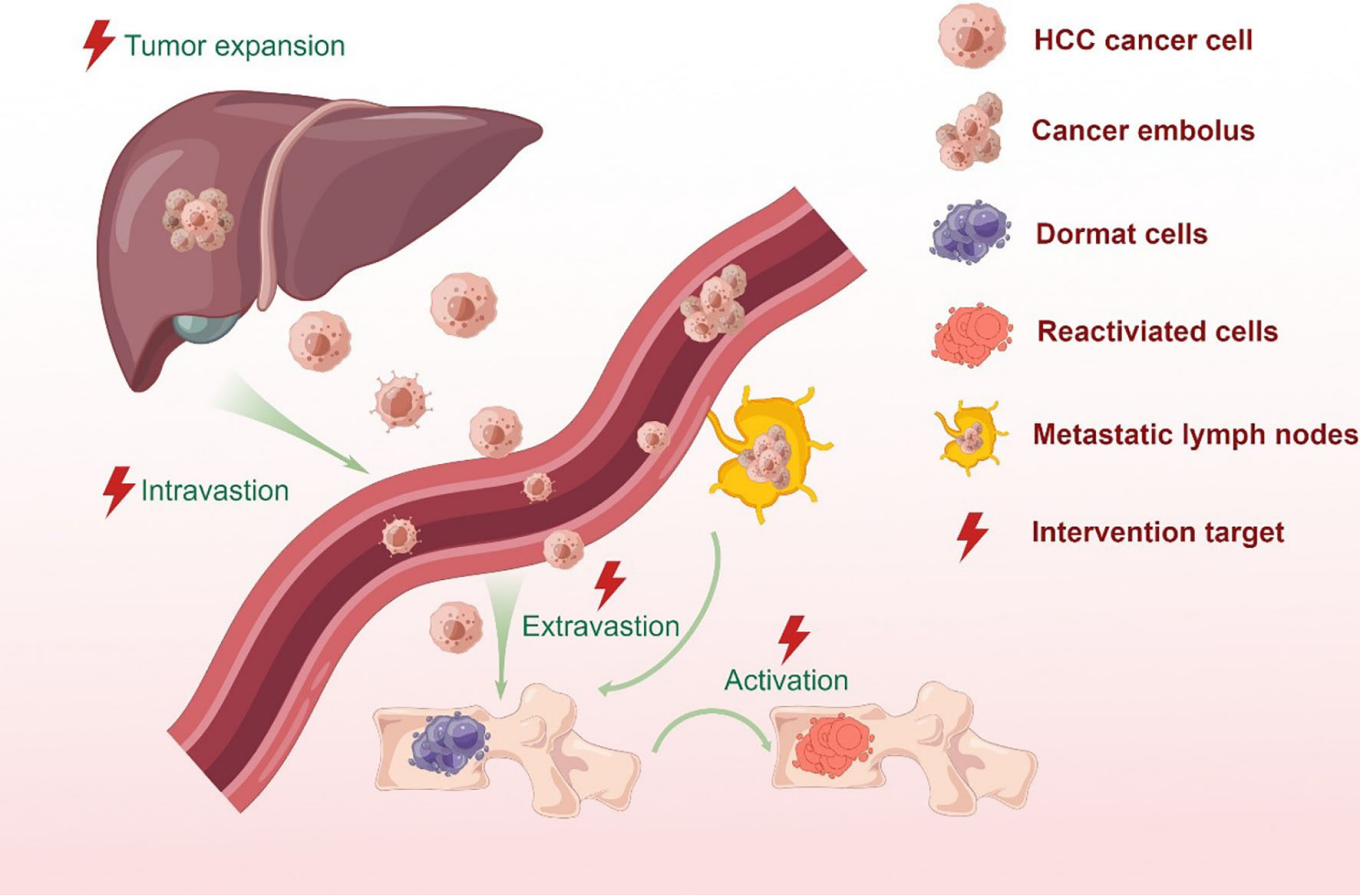
Bone metastatic steps of HCC cells. In situ hepatocellular carcinoma results from the mutation of protooncogenes or suppressor genes in normal cells as a combined result of physical, chemical or biological factors. Cancer cells stimulate angiogenesis to fulfill its nutritional needs. During tumor growth, the extracellular matrix is dissolved, which causes the invasion of the lymphatic and circulatory systems. But only a small proportion of cancer cells can survive and form a tumor thrombus in the circulatory system due to immune surveillance. Meanwhile, CTCs and ctDNA can be detected by the liquid biopsy for monitoring tumor in real time. At the proper time, the surviving cells migrate from the circulatory system to the target organ or tissue and colonize the area. Metastatic tumor cells in distant locations are inactive for a period of time in order to adapt to stress stimuli and survive a hostile environment. When stimulated by an appropriate signal, dormant tumor cells become active and continue to behave in a malignant fashion.

A small proportion of cancer cells in a primary HCC tumor have the metastatic potential to disseminate and distribute from the blood and/or lymphatic systems to distant tissues or organs (22) (See **Figure 1**). Various studies have demonstrated that both macro-and microvascular invasion are universally recognized as a predictor of distant metastasis for HCC (23, 24). The presence of tumor cells in the hepatic portal vein can contribute to intrahepatic metastasis, while they can also appear in the hepatic vein to induce the occurrence of distant metastasis of HCC and recurrence after liver transplantation. Clinicopathological features of HCC with portal hypertension account for the predilection of metastases to the spine throughout the valve-less venous plexus (25).

In recent years, the rapid development of radionics and artificial intelligence has made it easy to detect micro-vascular invasion (MVI) by analyzing the peritumoral area. This technology has unique diagnostic advantages and can guide HCC treatment selection and prognosis evaluation (24). Currently, the use of this technology for MVI detection is limited by the radiologists’ subjective judgment and lack of uniform guidelines. This method, however, is a promising way to determine the degree of HCC progression using simple imaging data before surgery. Cancer cells can also be found in neuronal spaces as well as the vascular and lymphatic systems, and the process of migration along nerves is termed perineural invasion (PNI) (26). After vascular invasion, metastatic cancer cells in the blood or lymph nodes are called circulating tumor cells (CTCs) and the presence of positive CTCs or ctDNA in HCC indicates a more aggressive phenotype and poorer prognosis (27, 28). Detection of CTCs and circulating tumor DNA (ctDNA) using the new emerging methodologies of liquid biopsy or lymph node biopsy is currently an important method for the diagnosis of metastatic cancers (29, 30).

### Preconditions for HCC-BM Establishment

The transport of cancer cells *via* blood vessels and lymph nodes, disseminates them from the primary tumor to bone marrow for further progression to bone metastasis (31). The CTCs enter the bone marrow compartment by crossing the endothelial cell barrier as well as the basement membrane of blood vessels, and engage in specialized microenvironments or ‘bone niche’. A few of these disseminated tumor cells (DTCs) survive to grow in the bone marrow after extravasating into the metastatic site (32). They can either exist as individual cells in a quiescent state or form small masses that do not expand. Besides the invasive qualities, they need to have the ability to adapt to the microenvironment in ways that encourage colonization (32). Often, DTCs that colonize bone and evade the immune system typically remain dormant for long periods. Cellular dormancy, moreover, is a profound mechanism that helps tumor cells evade immune surveillance and evolve to develop chemoresistance (33, 34).

The molecular mechanisms including extracellular matrix (ECM) development, metabolic and epigenetic changes, stemness, dysregulated non-coding RNAs, as well as the activation of the p38 stress-induced pathway, can mobilize dormant cancer cells to progress to form a micro-metastasis (35). As the metastasis progresses, the reactivated dormant cells proliferate uncontrollably and eventually begin to modify bone structure. Clinical speculation also indicates that these tumor cells are at their most vulnerable state in this transition stage for therapeutic targeting (36). Fortunately, technology has made it possible to study rare cells in bone, including dormant cells to complement our understanding of these early steps in BM development (36). Although the general progress of bone metastasis from different soft cancers is similar, however, the thorough and exact pathophysiology underlying the crucial early events of HCC-BM is not yet fully understood. Further research is necessary to elucidate the precise mechanism of DTCs in the bone and determine the optimum conditions for dormancy-break, or - eradication.

## HCC-BM Biomarkers

**Figure 2:**
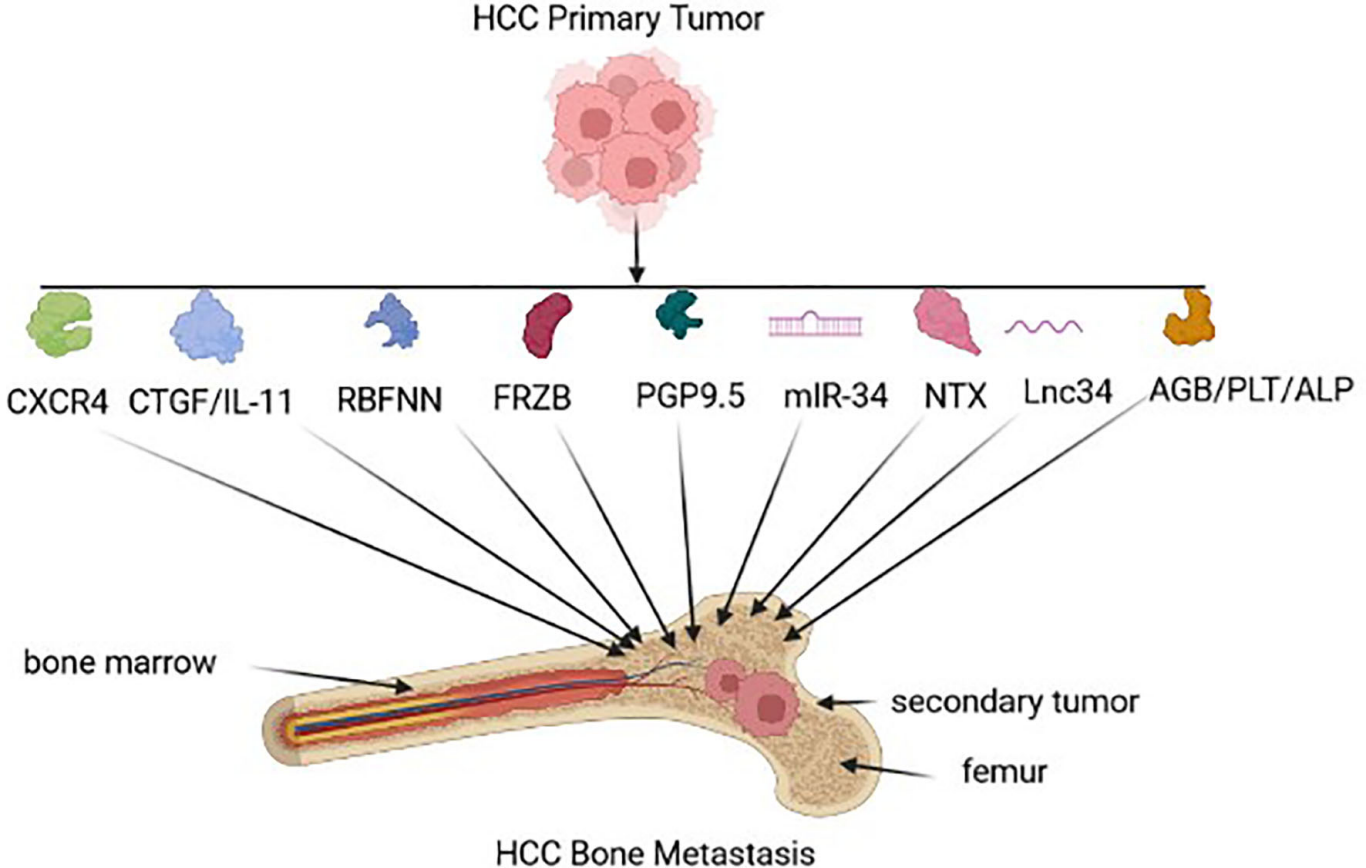
HCC biomarkers for Bone metastasis. Early HCC-BM biomarkers expressed by the tumor-environment include chemokine receptor type 4 (CXCR4), connective tissue growth factor (CTGF)/interleuken 11(Il-11), radial basis function neural network (RBFNN) algorithm containing six peptides, Secreted Frizzled Related Protein 3 (FRZB), neuron cytoplasmic protein 9.5 (PGP 9.5), microRNA-34a (miR-34a), N-terminal telopeptide (NTx), long non-coding RNA 34 (Lnc34), hemoglobin (HGB)/ platelet count (PLT), alkaline phosphatase (ALP).

According to various studies, HCC metastases and prognosis can be determined by reproducible gene expression patterns in the liver microenvironment (37, 38). Gene-expression profiles that strongly predict metastasis and poor outcome may already be present in primary tumors (see **Figure 2**). Potential biomarkers in serum, urine, and tumor tissue, that have been identified in a range of clinical studies to predict HCC-BM, as well as determine prognosis in HCC patients (see **Table 1**).

**Table 1:** Clinical studies about molecular markers in HCC-BM.

Year	Patient	Type of specimens	Biomarker	Status in HCC-BM	Clinical value	Refs
2009	HCC patients with/without BM (n = 43/138)	TMA	CXCR4 expression	Increase	An independent risk factor may be associated with poor clinical outcomes.	(39)
2011	HCC patients with/without BM (n = 24/24) and an independent cohort of 350 HCC patients which was conducted to evaluate the clinical significance of the candidate genes	FFPE, TMA	Intratumoral CTGF combined with IL-11 expression	Increase	An independent risk factor for HCC-BM	(40)
2014	HCC patients with/without BM (n = 66/72)	Serum	RBFNN model based on six significant peptides (m/z for these six peptides were 1535.4, 1780.7, 1866.5, 2131.6, 2880.4, and 2901.9)	Increase	A serological diagnosis tool for HCC-BM	(41)
2015	HCC patients with synchronous or metachronous BM received surgery (n=13)	FFPE	FRZB expression	Increase	A novel predictor for poor prognosis of HCC-BM after surgical resection	(42)
2015	HCC patients with sBM or mBM who received surgery (n=13)	FFPE	PGP9.5 expression	Increase	A potential role of the PNI in HCC-BM	(26)
2016	HCC patients with/without BM (n = 10/10) and an independent cohort of 106 HCC patients for evaluating candidate miRNAs and 296 HCC patients for evaluating the clinical significance of miRNA-34a	Serum, TMA	miRNA-34a expression levels in serum and intratumoral tissue	Decrease	An independent risk factor for HCC-BM	(43)
2017	HCC patients with BM who had been treated with ZOL (n=99)	Serum	The baseline serum NTX	Decrease	Reflecting longer progression-free survival	(44)
2021	HCC patients who underwent curative hepatectomy (n=157)	Serum	Circulating lnc34a expression	Increase	An independent risk factor for HCC-BM	(45)
2022	HCC patients with sBM or mBM (n=77/51)	Serum	Serum HGB, PLT and ALP level	Decrease/Decrease/Increase	Risk factors for HCC-BM.	(46)

IHC, immunohistochemistry; TMA, the tissue microarray; MALDI-TOF-MS, Matrix-assisted laser desorption ionization-time of flight mass spectrometry; LC-MS, liquid chromatography-mass spectrometry; m/z, Mass to charge ratio; RBFNN, radial basis function neural network; sBM, synchronous bone metastasis; mBM, metachronous bone metastasis; FRZB, frizzled-related protein; PGP9.5, protein gene product 9.5; PNI, perineural invasion; qRT-PCR, real-time quantitative polymerase chain reaction; ISH, in situ hybridization; ZOL, zoledronic acid; NTX, N-telopeptide of type I collagen; HGB, hemoglobin; PLT, platelet; ALP, alkaline phosphatase.

In HCC, the molecular pathogenesis is extremely complex and heterogeneous, and the process of metastatic dissemination from HCC can be generally separated into the sequential steps: primary tumor growth, cell polarity transition, seed, dormancy, and secondary outgrowth (47, 48). Each step cannot be missed, which means that the process of metastasis is time-consuming and complicated. Given the vulnerability of the metastatic process discussed above, a number of target-oriented HCC-BM biomarkers have been identified (see **Figure 1**/**Table 1**).

In primary HCCs, CXC chemokine receptor 4 (CXCR4) expression may be a biomarker for BM that is associated with poor clinical outcome (39). Stromal cell-derived factor-1 (SDF-1), also known as chemokine ligand 12 (CXCL12), can induce metastases through interacting with CXCR4. Interestingly, an elevated CXCR4 level alone does not necessarily result in HCC-BM due to the loss of SDF-1 expression (49). In another study, the expression of seven serum peptides sourced from AFP, prothrombin, fibrinogen beta chains, serglycin, transthyretin, isoform 2 of inter-alpha-trypsin inhibitor heavy chain H4 (ITIH4), and isoform 1 of autophagy-related protein 16-2 (ATG-16.2) were used as a collective predictive tool for HCC-BM detection (41). FRZB expression was also up-regulated in HCC-BM suggesting that FRZB might play a key predictor of HCC-BM (42). Neuronal cell bodies and axons of peripheral and central nervous systems express PGP9.5, which is a ubiquitin-protein hydrolase could also be considered as a BM biomarker. Interestingly, one study revealed that PGP 9.5 had a higher nerve density in bone metastases tissues compared with HCC tissues (26). The dysregulated expression of miR-34a as a serum and intra-tumoral tissue biomarker was also found to be a useful predictor for the risk of BM in HCC patients (43), as well as the elevated expression of lnc34a expression (45). In another serum based test, low baseline NTX levels could be correlated with a better outcome in HCC-BM patients with Child–Pugh grade A (44) and finally, serum HGB, ALP and PLT could also be useful predictors of BM-HCC versus HCC (46).

## Bone Remodeling and Bone Destruction

There is a balance between the resorption of mineralized bone by bone-resorbing cells (osteoclasts, OCs) and the formation of new bone by bone-forming cells (osteoblasts, OBs), which is essential for maintaining bone mass in humans (50). The process of bone remodeling is closely influenced by both systemic and local factors to maintain this physiological balance. Once bone metastasis initiation is activated by homotypic and heterotypic cellular interactions between cancer cells, OBs and OCs, normal bone homeostasis is easily broken and can lead to a negative chain reaction. Usually, osteolytic and osteogenic metastasis are two opposite types of cancer-related bone metastases (51). The classification is decided by which cell type exerts a dominant effect and represents two extremes of the dysregulation of bone homeostasis, which are bone lysis or sclerosis. Bone metastases in HCC are predominantly osteolytic in nature, and osteoblastic or mixed osteoblastic-osteolytic lesions are possible (21, 52). The main manifestations of osteolytic metastasis are an increased likelihood of systematic severe osteolytic bone lesions and SREs, resulting in fracture, nerve compression syndromes, and even paralysis (53–55). It is worth mentioning that, besides OBs and OCs, the bone microenvironment also consists of stromal cells, hematopoietic cells, and others. However, there is limited recent research reported on these important cells in the evolution of HCC-BM.

## Molecular Pathways of Bone Remodeling

As a key regulator of bone homeostasis, receptor activator of nuclear factor-kB (NF-κB) ligand (RANKL) is also referred to as osteoprotegerin ligand (OPGL), osteoclast differentiation factor (ODF), and TNF-related activation-induced cytokine (TRANCE). Various studies indicate that multinucleated osteoclasts originate from monocyte-/macrophage-lineage cells and resorb mineralized bone matrix by creating a microenvironment. Osteoclast precursors overexpress RANK when exposed to macrophage colony-stimulating factor (M-CSF), which binds to RANKL expressed by osteoblasts (56). Through the PI3K/Akt/mTOR and NF-κB pathway, the complex of RANK/RANKL recruits TNF receptor-associated factors (TRAF6), and finally induces osteoclast maturation (57, 58). In contrast, OPG, a soluble RANKL decoy receptor, could block its interaction with RANK by binding RANKL (59). The relative expression levels of RANKL and OPG determine the activation of osteoclasts and bone metabolism.

The normal bone microenvironment consists of osteoblasts, osteoclasts, stromal cells, mineralized bone matrix, hematopoietic cells, and others. But in the presence of metastatic HCC cells, its molecular interactions with the bone microenvironment disrupt the balance of bone metabolism and further drive skeletal metastases. Previous studies (60) have shown that metastatic tumor cells in many cancers including those of the breast, prostate and lung, could directly secret RANKL or induce osteoblasts to produce RANKL, thereby causing bone destruction and bone formation which is characterized by matrix degradation and release of numerous cytokines and growth factors. Sasaki et al. (61) firstly studied RANKL produced by HCC cells and showed that RANKL activated and differentiated osteoclast precursors, which resulted in osteolysis and bone metastasis. Parathyroid hormone-related protein (PTHrP), an important prognostic factor of malignancy-associated hypercalcemia (MAH), was known to increase the RANKL expression and repress the OPG expression, indicating that PTHrP could promote bone metastasis in breast cancer (62–64). Previous studies have shown that a similar expression trend of PTHrP is undetectable in HCC cells by immunohistochemistry (65). On the other hand, recent clinical studies addressing PTHrP-mediated hypercalcemia have demonstrated that PTHrP can be detected in HCC patients from Australia and Korea (66, 67). Despite the important role of PTHrP in calcium/phosphorus metabolism and bone resorption, there has been no previous evidence of a link between PTHrP expression and bone metastasis in HCC, unlike in other cancers.

## Possible HCC-BM Therapeutic Pathways

Due to the absence of obvious symptoms in the early onset of bone metastases, the diagnosis of bone metastases is difficult, and an effective treatment has not yet been established. The presence of multiple levels of intratumor heterogeneity is recognized as a distinct characteristic of human tumors that modulates the efficacy of targeted therapy in HCC-BM (see **Table 2**). Based on next-generation sequencing (NGS), however, important driver of mutations in HCC cells have been determined including EGFR, ALK, KRAS, and PI3KCA (72).

**Table 2:** Recent advances in molecular mechanisms and oncogenes in HCC-BM.

Target	Clinical association	Location	Main mechanism	Blocking method	Refs
LGALS3	LGALS3- overexpression promotes HCC bone metastasis	The outermost covering of OP cells	Promoting differentiation and Activation of Osteoclasts by activating CD98- and integrin αv/β3 complex-mediated fusion and podosome formation	LGALS3 neutralizing antibody or YAP inhibitor verteporfin	(68)
H19	H19-overexpression promotes HCC bone metastasis	Chromosome 11p15.5	Reducing OPG expression *via* PPP1CA-induced P38 dephosphorylation process and promoting ZEB1-dependent EMT by downregulating miR-200b-3p	P38 inhibitor	(69)
LncZEB1-AS1	LncZEB1-AS1-overexpression promotes HCC bone metastasis	Chromosome 10p11.22 region contiguous with ZEB1	Inducing MMP2, MMP7 and MMP9 upregulation *via* inhibiting miR-302b, resulting in enhanced EGFR/PI3K-AKT signaling	Compounds targeting the lncZEB1-AS1‐miR-302b-EGFR axis	(70)
Lnc34a	Lnc34a-overexpression promotes HCC bone metastasis	Enriching in colon cancer stem cells (CCSCs)	Recruiting Dnmt3a *via* PHB2 and HDAC1 to methylate and deacetylate the miR-34a promoter and thereby targeting Smad4 *via* the TGF-β pathway	Increasing miR-34a or decreasing Smad4	(71)

The secreted multifunctional glycoprotein LGALS3 (Lectin galactoside-binding soluble 3) is generally upregulated in tumors through various intracellular and extracellular mechanisms (73). LGALS3 produced by HCC is located on the outer surface of osteoclast progenitor cells (OPs) and promotes CD98- and integrin 𝛼α/𝛽β complex–mediated fusion and podosome formation of osteoclasts. This is explained by increased the formation of TRAP^+^-multinuclear OCs and TRAP activity, but no alteration of ALP^+^-OBs along with the interface of bone-tumor (68). Furthermore, E3 ligase RNF219-mediated α-catenin degradation promotes the overexpression of LGALS3 through YAP1/β-catenin-dependent epigenetic modifications, resulting in HCC-BM and following SREs. A potential therapeutic option, namely, Verteporfin (VP), a YAP specific inhibitor, can block RNF219/α-catenin/LGALS3 axis to effectively inhibit HCC-BM (74). Other studies have focused on the role of long non-coding transcripts in HCC-BM. Long noncoding RNAs (lncRNAs) are RNAs with limited or no coding potential that are longer than two hundred nucleotides in size (75). Emerging evidences indicated that dysregulated lncRNAs lead significantly to the initiation and progression of tumorigenesis (76). Targeting dysregulated lncRNA in HCC-BM pathways, thus, offers another therapeutic option. It has been suggested that the expression of lncZEB1-AS1, an oncogenic lncRNA, is evident in HCC patients with lung (77) and bone metastasis (70). Zinc finger E-box binding homeobox 1 (ZEB1) is also a pivotal regulator that induces EMT and metastasis. LncZEB1-AS1, however, also influences matrix metalloproteinases (MMPs) without EMT-related markers including Vimentin, N-cadherin, or E-cadherin (70). Furthermore, lncZEB1-AS1 serves as a ceRNA for miR-302b and thereby promotes tumor progression *via* EGFR-PI3K-AKT signaling although no clear evidence about the effect of lncZEB1-AS1 on HCC-BM has been directly demonstrated (70). Lnc34a expression in serum is a potential predictive biomarker for HCC-BM patients (45). Previous series studies indicated that lnc34a epigenetically inhibits miR-34a expression through recruiting DNMT3a *via* PHB2 to methylate miR-34a promoter, as well as HDAC1 to promote histones deacetylation. Alternatively, miR-34a also targets Smad4 through the TGF-β pathway and then alters transcription of downstream genes related with BM, for instance, CTGF and IL-11 (71).

## Summary

HCC metastasizes into bone tissue after a cascade of short-term responses. Genetic factors, the tumor microenvironment (TME), and other factors all work in concert to promote HCC-BM. Developments in diagnostic technologies, however, allow us to detect HCC-BM in earlier stages using multiple novel technologies, including medical image analysis which is based on artificial intelligence (AI), as well as the use of liquid biopsies and next-generation sequencing (NGS). The accurate detection of HCC-BM stage and its molecular features also promote a clearer picture for patient treatment and prognosis. Currently, traditional treatments such as surgery, radiotherapy and chemotherapy, as well as targeted therapy including sorafenib and bisphosphonate, remain complicated by the complex nature of HCC-BM. However, future research that promotes a better understanding of the intra-tumoral heterogeneity of HCC-BM can improve the development of improved therapy. Understanding the key to heterogeneity will help us develop individualized approaches for HCC patients and new generation drugs can be adjusted accordingly. Alternatively, this understanding can promote the better deployment of current drug alternatives at different stages of HCC-BM pathogenesis. However, our understanding of the molecular etiology of the HCC-BM tumor microenvironment needs to be improved by further studies covering energy metabolism and differential immune responses. In summary, we have offered some novel perspectives in HCC-BM showing that it is a complex and multistep process that currently leads to mortality. Understanding the TME and HCC-BM mechanisms will help drive future potential therapeutic interventions as a way to improve the quality of life and extend the lives of HCC-BM patients.

## Data Availability Statement

The original contributions presented in the study are included in the article/supplementary material. Further inquiries can be directed to the corresponding authors.

## Author Contributions

XY, MZ, and XZ reviewed the literature and wrote the manuscript. DC, JL, and DS revised the manuscript. XQ, YL, and KS reviewed, revised the manuscript and performed figures and tables of the manuscript. All authors contributed to read and approved the submitted version.

## Funding

This work was supported by the National Natural Science Foundation of China (81971504), Post-Doctoral Special Foundation of China (2020M670065ZX), Post-Doctoral Foundation of Jiangsu Province (2020Z021), Changzhou Society Development Funding (CE20205038), The lifting Project of 2021 Young Scientific and technological talents in Changzhou, Youth Science and Technology Talent Program of Changzhou Health and Family Planning Commission (QN202106 and QN202007), Changzhou Sci&Tech Program (CJ20210085), and Youth Talent Development Plan of Changzhou Health Commission (CZQM2020047).

## Conflict of Interest

The authors declare that the research was conducted in the absence of any commercial or financial relationships that could be construed as a potential conflict of interest.

## Publisher’s Note

All claims expressed in this article are solely those of the authors and do not necessarily represent those of their affiliated organizations, or those of the publisher, the editors and the reviewers. Any product that may be evaluated in this article, or claim that may be made by its manufacturer, is not guaranteed or endorsed by the publisher.
